# Spatial Differences of Ecological Well-Being Performance in the Poyang Lake Area at the Local Level

**DOI:** 10.3390/ijerph191811439

**Published:** 2022-09-11

**Authors:** Shengyun Wang, Liancheng Duan, Qin Zhu, Yaxin Zhang

**Affiliations:** 1 Research Center of the Central China for Economic and Social Development, Nanchang University, Nanchang 330031, China; 2 School of Economics and Management, Nanchang University, Nanchang 330031, China; 3 School of Public Policy and Administration, Nanchang University, Nanchang 330031, China

**Keywords:** ecological well-being performance, two-stage Super-NSBM model, DEA window analysis, Dagum Gini coefficient, spatial differences

## Abstract

Maintaining low ecological consumption and high well-being while making economic progress and thus achieving sustainable development is a significant issue facing the world today. Ecological well-being performance (EWP) is one indicator that tracks this progress. Although EWP has been typically assessed at the national, provincial and urban levels, investigations into EWP units in the Great Lakes region are lacking. This study applied the two-stage super-efficiency slack-based measure (Super-NSBM) model and DEA window analysis to evaluate the EWP and sub-stage efficiency in the Poyang Lake (largest freshwater lake in China) area and analyzed its spatial differences at the local level. Redundancy analysis was conducted to explore the EWP improvement paths of different counties in the Poyang Lake region and Dagum’s Gini coefficient was applied to clarify the policy priorities of coordinated regional development. Results showed that the EWP of the Poyang Lake area presents an improving trend from 2007 to 2019, although the overall level is still low. The Poyang Lake area mainly faces the incongruity of ecological and economic development, which is the main reason for the low overall level of EWP. Excessive resource input and severe environmental pollution are common in most counties, but the focus of measures to improve EWP differs from county to county. The expansion of spatial differences in eco-economic efficiency leads to the spatial differences of EWP in the Poyang Lake area being still apparent at the local level. Reducing inter-basin disparities while alerting the widening of intra-basin differences is the policy focus for the future coordinated development of the Poyang Lake area.

## 1. Introduction

Achieving economic prosperity and social progress within ecological limits is the essential requirement of sustainable development for improving human well-being [1]. Daly (1996) [2] pointed out that the role exhibited by economic growth in this process is only the means to an end but not an ultimate end. Once the ecological environment is damaged, the lack of ecological services will harm human well-being [3]. In this sense, a healthy ecology is a basis for enhancing human well-being [4]. The Great Lakes region is an essential part of the ecosystem with ecological functions, such as maintaining species diversity, regulating climate and degrading pollution [5]. Coordinating the relationship among environmental protection, economic progress and social development in the Great Lakes region can help promote the sustainable enhancement of human well-being [6]. The American economist Daly (1974) [7] first proposed EWP, which refers to the increase in the level of well-being per unit of ecological consumption. EWP has unique advantages in assessing the degree of coordinated ecological, economic and social development and is widely used in national or regional sustainable development studies [8].

Poyang Lake is located in the north of Jiangxi Province in China. Its annual average water volume accounts for about 15.6% of the runoff of Yangtze River and it is the largest freshwater lake in China [9]. Poyang Lake records more than 400 kinds of birds and 90 kinds of fish and the lake covers an area of 3150 square kilometers, which is crucial to the ecological pattern of China and even the world. Meanwhile, the Poyang Lake area has created more than 60% of total economic volume. with 30% of the province’s area. It has become the leading area of economic development in Jiangxi Province [10]. Accurately measuring the EWP of the Poyang Lake area to coordinate the relationship between economy, ecology and society can help the Great Lakes region play an exemplary role in China’s high-quality economic development. In addition, according to the theory of unbalanced regional development, the differences of local administrative units in resource endowment and development stage are likely to lead to spatial differences in EWP in the Poyang Lake area. Therefore, analyzing the spatial differences of EWP in the Poyang Lake area at the local level is also a significant focus of this study.

## 2. Literature Review

The American economist Daly (1974) [7] first expressed EWP as “service/throughput”. Service refers to the utility humans obtain from ecosystems. Throughput refers to the low-entropy energy and materials humans obtain from ecosystems and the high-entropy waste discharged into ecosystems. However, usage of the concept of EWP has been limited due to the lack of indicators that precisely quantify well-being output and ecological consumption. Zhu (2013) [11] introduced the concept of EWP in China and expressed it as the ratio of human development index (HDI) to ecological footprint (EF) that broadens its scope for development. In essence, EWP measures eco-efficiency in terms of enhancing well-being [12]. Therefore, EWP is an extension of sustainable development, broader than eco-efficiency.

In recent years, scholars have successfully conducted numerous sustainable development studies through EWP. They have mainly used two methods to evaluate EWP, namely, ratio and the efficiency model [12]. HDI [13] and life expectancy at birth (LEB) [14] are commonly used to measure objective well-being outputs and life satisfaction [15] as well as happiness index [16], commonly used to measure subjective well-being outputs in studies that apply the ratio method. EF is a commonly used indicator for measuring ecological consumption, with characteristics and salient advantages in calculating ecological consumption in terms of consumption and both source and sink dimensions [17]. Feng et al. (2019) [18] applied the ratio of HDI to EF to measure EWP in 30 Chinese provinces from 1994 to 2014 and examined the effects of industrial restructuring green (ISGA) and green total factor productivity (GTFP) on EWP growth. Common et al. (2007) [19] combined LEB with life satisfaction and measured the level of sustainable development in 143 countries using the ratio of happy life years (HLY) to EF. Behjat and Tarazkar (2021) [20] applied the ratio of HDI to EF to measure EWP in Iran and the results showed a decreasing trend of EWP in Iran from 1994 to 2004.

Some scholars have argued that the ratio method presents two limitations [21]. On the one hand, a single indicator cannot fully reflect ecological consumption or well-being output. On the other hand, the large magnitude of EF changes leads to the EWP measurement results being easily dominated by EF changes. Therefore, the researchers used an efficiency model approach based on multiple inputs and outputs. Wang et al. (2021) [22] used a super-efficient SBM model to measure the EWP of the Yangtze River Economic Zone from 2007 to 2017. Xiao and Zhang (2019) [23] applied an improved SFA model to analyze spatial and temporal characteristics of green innovation efficiency and EWP coupling and coordination in 30 Chinese provinces from 2004 to 2015. However, uniform rules for indicator selection are unavailable given that the efficiency model approach requires the identification of multiple input and output indicators. Bian et al. (2020) [24] utilized resource consumption and environmental pollution as input indicators and the three elements of HDI (income, education and health) as output indicators of well-being, as well as the Super-SBM model, to measure the EWP of 30 provincial capitals in China. Guo et al. (2022) [25] measured the EWP of the Yangtze River Economic Zone from 2004 to 2018 by using resource consumption as an input indicator and the three elements of the Human Development Index and environmental pollution as output indicators.

In addition, regional differences are a popular part of EWP research and have been studied extensively by scholars. For example, Wang et al. (2021) [8] applied the Dagum Gini coefficient to analyze the spatial differences among the eight economic regions in China and the findings suggest that reducing inter-regional differences is the key to alleviating regional imbalances in China. Bian et al. (2019) [26] applied the Super-SBM model to assess the EWP of 278 cities in China from 2005 to 2016. They found that the EWP of cities in the eastern region was significantly higher than those in the central and western areas. Fang et al. (2019) [27] applied the Moran index to analyze the spatial effects of 30 provinces in China and the results of the study showed that the EWP of each province was in a solid positive spatial correlation.

Although EWP has been successfully applied, it can still improve in the following aspects. First, most studies on spatial differences in EWP have been conducted under the large-scale levels of regions, provinces and cities and there is a lack of spatial difference analysis at the local level of the Great Lakes region. Second, studies typically consider ecological inputs but ignore nonecological inputs, such as scientific and technological expenditures and human capital inputs, in the process of indicator selection. These elements are indispensable intermediate means of converting ecological consumption to human well-being. Finally, existing studies only consider EWP as a simple direct conversion process from ecological consumption to well-being output and lack the analysis of sub-stage efficiency.

Compared with previous studies, the main objectives of this study are presented as follows. First, nonecological inputs, such as science and technology expenditure and human capital input, are included in the evaluation index system to assess the level of EWP in the Poyang Lake area comprehensively and accurately. Second, the spatial differences of EWP and its sub-stage efficiency in the Poyang Lake area at the local level are analyzed and the Dagum Gini coefficient is used to explore the characteristics and sources of spatial differences. Third, the redundancy analysis is used to reveal what measures each county should take to enhance EWP. We hope that the analysis of this study can provide scientific policy reference for the local level of the Poyang Lake area to enhance EWP in a coordinated manner.

## 3. Materials and Methods

### 3.1. Materials

#### 3.1.1. Division of Regions

**Figure 1:**
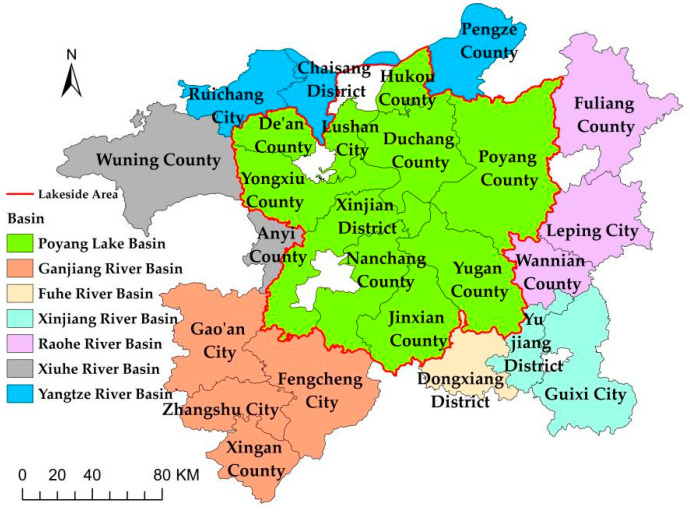
The regional division of the Poyang Lake area.

The specific study area identified in this thesis includes 25 counties (districts or cities) within the Poyang Lake area, given the availability of data. Notably, Yujiang County was renamed Yujiang District in 2018, Jiujiang County was renamed Chaisang District in 2017, Dongxiang County was renamed Dongxiang District in 2016, Xingzi County was renamed Lushan City in 2016 and Xinjian County was renamed Xinjian District in 2015. However, this adjustment only changes the name and district boundaries remain the same. Names of administrative divisions after the adjustment are used in this study to ensure uniformity in presentation. The two main regional classification methods for the Poyang Lake area in the literature are “center–periphery” [28] and “by basin” [29]. We integrated these two methods and adopted the regional division method of “lakeside–periphery–basin” in this work to analyze the EWP of the Poyang Lake area. The specific division scheme is presented in Figure 1.

The Poyang Lake area is divided into two parts: the lakeside area and the peripheral area around the lake. Poyang Lake basin belongs to the lakeside area and Ganjiang River basin, Xinjiang River basin, Raohe River basin, Xiuhe River basin, Yangtze River basin and Fuhe River basin belong to the peripheral area around the lake.

#### 3.1.2. EWP Analysis Framework and Indicator Selection

**Figure 2:**
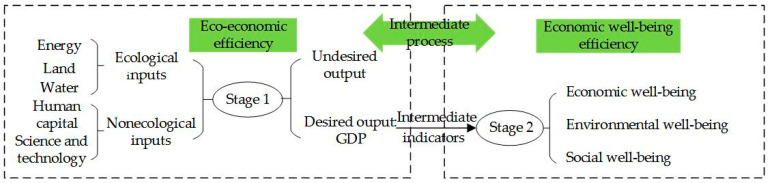
Eco-economic efficiency and economic well-being efficiency transformation system of EWP in China.

On the basis of Long (2019) [30] and Wang et al. (2021) [31], the present study decomposed EWP into two stages (Figure 2): (1) nonecological inputs (including human capital and science and technology expenditures) are used as intermediate means and ecological inputs (including energy, land and water) are transformed into undesired (environmental pollution) and desired (economic outputs) outputs and (2) intermediate indicators (economic outputs) are converted as inputs of integrated economic, environmental and social aspects of well-being outputs. The first stage, involving the conversion of ecological inputs into economic outputs, refers to eco-economic efficiency. The second stage, involving the transformation of economic inputs into integrated well-being outputs, refers to economic well-being efficiency.

The evaluation index system of EWP in the Poyang Lake area is presented in Table 1. On the basis of Wang et al. (2021) [8], the present study divided input indicators into ecological and nonecological inputs. Energy, land and water consumption are considered important indicators of ecological inputs [32,33,34]. On the basis of previous studies, this work uses energy consumption per capita (standard coal) estimated from nighttime lighting data to measure energy consumption [35]. Land resource consumption was measured by the built-up area per capita [24]. Water use per capita covers agricultural, industrial, domestic and ecological water use data and is utilized to measure water consumption [23,24]. Zhang et al. (2018) [36] defined EWP as the efficiency of the process of transforming ecological consumption to the level of human well-being. The human processing of resource inputs into actual products that increase the level of human well-being requires elements, such as science, technology, human capital and other inputs, as its intermediate means in this procedure [26]. Therefore, human capital, science and technology expenditures are used in this work to represent nonecological inputs. Among them, human capital and science and technology inputs are measured using education expenditure per capita and science and technology expenditure per capita, respectively.

Output indicators of the first stage also include both desired and undesired outputs. If the process of converting ecological inputs into economic outputs results in environmental pollution, then it can harm public health [37]. Therefore, this study uses environmental pollution to represent the undesired output. According to existing studies [26], the top representative indicators for measuring environmental pollution are wastewater, waste gas and waste residue. However, due to the lack of data on waste gas and waste residue in the Poyang Lake area, we selected other representative indicators to measure environmental pollution. We used the per capita wastewater discharge indicator to measure water pollution and the accuracy of this indicator has been confirmed in studies by Xiao and Zhang (2019) [23] and Bian et al. (2020) [24]. By reviewing the China Statistical Yearbook, we found that particulate matter is the main component of pollutants in waste gas in Jiangxi Province. Therefore, we selected the annual average PM2.5 concentration to measure air pollution. In addition, in view of China’s explicit “dual carbon target” of peak carbon and carbon neutrality in 2020, we selected a per capita carbon emission indicator to measure the environmental pollution caused by carbon emissions, referring to the study of Wang et al. (2021) [8]. We selected the representative GDP per capita indicator for the desired output to measure economic output. Meanwhile, GDP per capita is also an intermediate indicator, that is, the economic input in the second stage.

HDI has been commonly used to measure the well-being output in existing studies. However, sub-indicators of the HDI cover only economic and social dimensions but ignore the importance of environmental well-being. Xiao and Xiao (2021) [38] incorporated environmental well-being into the evaluation index system to measure EWP in cities in the Yellow River basin. Ibrahim et al. (2021) [39] integrated environmental performance into the evaluation index system of EWP to examine socio-ecological efficiency in sub-Saharan African countries. Therefore, this study measures well-being outputs using a composite well-being that includes economic, environmental and social dimensions. Forest coverage rate, energy saving and environmental protection expenditure reflect the level of regional ecological construction. Sewage treatment rate measures the ability of the region to deal with water environment pollution. Meanwhile, carbon dioxide sequestration measures the level of carbon sequestration through terrestrial vegetation and the ability to reduce carbon dioxide emissions in each county and district as well as reflecting the power of the region, to a certain extent, to improve environmental quality. Therefore, these four indicators are selected to reflect the environmental well-being of regional residents in this study. Urban and rural per capita disposable income is a reasonable indicator of economic well-being in terms of the economic dimension. Meanwhile, indicators at the education, health and social security levels are selected to measure social well-being in this study. Well-being at the education level is measured through the average number of years of education. Health-level well-being is measured via the number of skilled health personnel per 10,000 people. Well-being at the social security level is measured using the public expenditure per capita, social security and employment expenditure per capita and number of employed persons.

Notably, the indicators of environmental pollution and total well-being output in this study are selected in large numbers, while the number of decision making units (DMUs) is small. This study adopts the entropy value method to accurately measure the EWP of each county (district or city) within the Poyang Lake area and reduce the dimensionality of environmental pollution and total well-being output. Finally, the indices of environmental pollution, economic well-being and social well-being are obtained.

#### 3.1.3. Data Sources

Data for the selected indicators are extracted from the 2008–2020 China County and the Jiangxi Provincial Statistical Yearbooks. Data for average years of education are obtained from the sixth and seventh national census bulletins published by the government of each county (district or city). Data on nighttime lighting were obtained from the Harvard Dataverse platform [40]. Data for wastewater discharge are obtained from China County Construction Statistical Yearbook. Data on average PM2.5 concentrations are from the University of Washington’s Atmospheric Composition Analysis Group [41]. In addition, data on carbon emissions and carbon dioxide sequestration were obtained from the China Carbon Accounting Database [42] and missing data for individual years were supplemented via interpolation.

We calculated the EWP of 25 counties (districts or cities) in the Poyang Lake area using MAXDEA 6.0 software. We then applied MATLAB R2015b (MathWorks, Natick, MA, USA) to estimate spatial differences of EWP in the Poyang Lake area.

### 3.2. Methods

#### 3.2.1. EWP Evaluation Model: Two-Stage Super-NSBM Model and DEA Window Analysis

This study adopted a two-stage Super-NSBM model [30] to measure EWP in the Poyang Lake area and open EWP’s “black box” in converting ecological consumption into well-being output. ρ*se represents EWP. EWP and its sub-stage efficiency in the Poyang Lake area are calculated as follows:

(1) $$\rho_{\mathrm{se}}^{*}=\min\frac{\sum_{k=1}^{K}w^{k}\left[1+\frac{1}{m_{k}}\left(\sum_{i=1}^{m_{k}}\frac{s_{i}^{k-}}{x_{i0}^{k}}\right)\right]}{\sum_{k=1}^{K}w^{k}\left[1-\frac{1}{v_{1k}{+v}_{2k}}\left(\sum_{r=1}^{v_{1k}}\frac{s_{r}^{\mathrm{gk}}}{y_{r0}^{\mathrm{gk}}}+\sum_{r=1}^{v_{2k}}\frac{s_{r}^{\mathrm{bk}}}{y_{r0}^{\mathrm{bk}}}\right)\right]}\;\;s.t.\;\left\{\begin{array}{c} \sum_{j=1,j\neq 0}^{n}x_{ij}^{k}\lambda_{j}^{k}+s_{i}^{k-}=\theta^{k}x_{i0}^{k},i=1,\cdots ,m_{k},k=1,\cdots ,K\\ \sum_{j=1,j\neq 0}^{n}y_{rj}^{gk}\lambda_{j}^{k}+s^{gk}=\varphi^{k}y_{r0}^{gk},r=1,\cdots ,s_{k},k=1,\cdots ,K\\ \sum_{j=1,j\neq 0}^{n}y_{rj}^{bk}\lambda_{j}^{k}-s^{bk}=\delta^{k}y_{r0}^{bk},r=1,\cdots ,s_{k},k=1,\cdots ,K\\ \varepsilon \leq 1-\frac{1}{v_{1k}+v_{2k}}\left(\sum_{r=1}^{v_{lk}}\frac{s_{r}^{gk}}{y_{r0}^{gk}}+\sum_{r=1}^{v_{2k}}\frac{s_{r}^{bk}}{y_{r0}^{bk}}\right)\\ z^{\left(k,h\right)}\lambda^{h}=z^{\left(k,h\right)}\lambda^{k},\sum_{j=1,j\neq 0}^{N}\lambda_{j}^{k}=\sum_{k=1}^{K}w^{k}=1\\ \lambda^{k}\geq 0,s^{k-}\geq 0,s^{gk}\geq 0,w^{k}\geq 0 \end{array}\right.$$

where *m_k_* and *v_k_* represent the input and output of the stage *k*, respectively; *φ^k^* represents the number of intermediate indicators; (*k*,*h*) represents the connection from stage *k* to stage *h*; *x*, *y*, *z* and represent the input, output and intermediate output, respectively; *λ^k^* represents the model weight of stage *k*; and *ω^k^* represents the weight of stage *k*; *s^k−^* represents the slack variables of input indicators; and *s^gk^* and *s^bk^* represent the slack variables of desired and undesired outputs, respectively.

The DEA window analysis was adopted in this study to measure EWP in the Poyang Lake area and analyze differences in EWP and trend of efficiency changes in each county (district or city) comprehensively. This method first treats the same DMU in varying periods as different DMUs for efficiency calculation. The efficiency of each year in other windows is then weighted and averaged to obtain the final efficiency value of the evaluated DMU. Notably, this method can increase the number of DMU and compare the efficiency in both horizontal and vertical dimensions [43]. On the basis of Cullinane et al. (2004) [44], we set the window width to 3 in this study.

#### 3.2.2. Spatial Differences Measurement: Dagum Gini Coefficient Decomposition

The Dagum Gini coefficient method improved the shortcomings of the traditional methods of measuring spatial differences and solved the problem of cross-over between samples [45]. It can not only quantitatively measure the magnitude of spatial differences but also reveal the sources of spatial differences. In this method, the overall Gini coefficient (*G*) of EWP in the Poyang Lake area is decomposed into intra-regional (intra-basin) differences (*G_jj_*), inter-regional (inter-basin) differences (*G_jh_*) and super-variable density (*G_t_*) and satisfy: *G* = *G_jj_* + *G_jh_* + *G_t_*.

The formula for measuring the Dagum Gini coefficient can be expressed as follows:

(2) $$G=\frac{\sum_{j=1}^{k}\sum_{h=1}^{k}\sum_{i=1}^{n_{j}}\sum_{r=1}^{n_{h}}/y_{\mathrm{ji}}-y_{\mathrm{hr}}/}{2\cdot \mu \cdot n^{2}}$$

where *μ* represents the mean value of EWP in each region, *n* represents the number of counties (districts or cities) in Poyang Lake area, *k* represents the number of regions, *n_j_*(*n_h_*) represents the number of counties (districts or cities) in the *j*(*h*) region (basin) and *y_ji_*(*y_hr_*) represents the EWP of the *i*(*r*) counties (districts or cities) in the *j*(*h*) region (basin).

(3) $$G_{w}=\overset{k}{\underset{j=1}{\sum }}G_{\mathrm{jj}}\cdot p_{j}\cdot s_{j}$$

(4) $$G_{b}=\overset{k}{\underset{j=2}{\sum }}\overset{j-1}{\underset{h=1}{\sum }}G_{\mathrm{jh}}\cdot {(p}_{j}\cdot S_{h}{+p}_{h}\cdot S_{j})\cdot D_{\mathrm{jh}}$$

(5) $$G_{t}=\overset{k}{\underset{j=2}{\sum }}\overset{j-1}{\underset{h=1}{\sum }}G_{\mathrm{jh}}\cdot {(p}_{j}\cdot S_{h}{+p}_{h}\cdot S_{j})\cdot {(1-D}_{\mathrm{jh}})$$

(6) $$G_{\mathrm{jj}}=\frac{\sum_{i=1}^{n_{j}}\sum_{r=1}^{n_{j}}\left|y_{\mathrm{ji}}-y_{\mathrm{jr}}\right|}{2\cdot \mu_{j}\cdot n_{j}^{2}}$$

(7) $$G_{\mathrm{jh}}=\frac{\sum_{i=1}^{n_{j}}\sum_{r=1}^{n_{h}}\left|y_{\mathrm{ji}}-y_{\mathrm{hr}}\right|}{{(\mu }_{j}{+\mu }_{h})\cdot n_{j}\cdot n_{h}}$$

(8) $$D_{\mathrm{jh}}=\frac{d_{\mathrm{jh}}{-e}_{\mathrm{jh}}}{d_{\mathrm{jh}}{+e}_{\mathrm{jh}}}$$

(9) $$d_{\mathrm{jh}}=\int_{0}^{\infty }{\mathrm{dF}}_{j}\left(y\right)\int_{0}^{y}{(y-x)\mathrm{dF}}_{h}\left(x\right)$$

(10) $$e_{\mathrm{jh}}=\int_{0}^{\infty }{\mathrm{dF}}_{h}\left(y\right)\int_{0}^{y}{(y-x)\mathrm{dF}}_{j}\left(y\right)$$

(11) $$p_{j}=\frac{n_{j}}{n},\;s_{j}=\frac{n_{j}\cdot \mu_{j}}{n\cdot \mu }$$

where *G_jj_* denotes the Dagum Gini coefficient of the *j*-th region (basin), *G_jh_* denotes the inter-regional (inter-basin) Dagum Gini coefficient of the *j*-th and *h*-th regions, *D_jh_* denotes the relative impact of EWP between the *j*-th and *h*-th regions (basins), *F_j_*(*F_h_*) is the cumulative density distribution function of the *j*-th (*h*-th) region (basin) and *d_jh_* denotes the differences of EWP between regions (basins). The Dagum Gini coefficient varies between 0 and 1. Spatial differences of EWP among counties (districts or cities) in the Poyang Lake area are small as the value of the Dagum Gini coefficient approaches 0 and vice versa.

## 4. Results

### 4.1. Spatial and Temporal Pattern Evolution of EWP in the Poyang Lake Area

#### 4.1.1. Time-Series Evolution of EWP in the Poyang Lake Area

**Figure 3:**
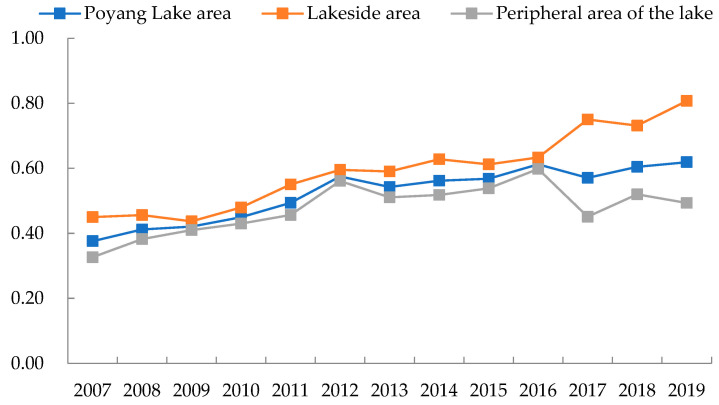
Time-series trends of EWP in the Poyang Lake area in the period of 2007–2019.

Figure 3 reflects the temporal evolution trend of EWP in the Poyang Lake area. The EWP of the Poyang Lake area improved from 0.376 in 2007 to 0.619 in 2019, with an average annual growth rate of 4.240%. The improving overall trend of EWP indicated that the efficiency of ecological consumption into the comprehensive well-being level in the Poyang Lake area has improved significantly. The overall level of EWP in the lakeside area was higher than that of peripheral area of the lake and the average annual growth rate is higher. In contrast, EWP’s average annual growth rate in the peripheral area of the lake is only 3.497%. The gap in EWP between the lakeside area and the peripheral area of the lake has been gradually widened and by 2019 it is already quite evident.

EWP is still at a low level from the overall perspective despite its rapid improvement in the Poyang Lake area (Table 2). The average value of EWP in all seven basins of less than 1 fails to realize the relative effectiveness of DEA. The annual average value of EWP in the Raohe River basin reaches the maximum, with an average yearly growth rate of 5.619%, due to the progress of Guixi City and Wannian County in ecological civilization construction. By contrast, the level of well-being in the Fuhe River basin progressed slowly, with an annual average value of EWP of only 0.239.

Specifically, among 25 counties (districts or cities) in the Poyang Lake area, only Duchang County, Poyang County and Gao’an City demonstrate a decreasing trend of EWP, thereby indicating that the overall EWP in the Poyang Lake area is improving. Among them, Nanchang County, De’an County, Xinjian District and Yongxiu County rank in the top four in terms of annual growth rate at 16.453%, 14.089%, 13.183% and 13.067%, respectively, and all achieved the relatively effective DEA. However, the overall level of EWP in the Poyang Lake basin is pulled down due to the low EWP in Lushan City, Xinjian District and Hukou County and the decreasing trend of EWP in Duchang and Poyang Counties. Similar to that of Duchang and Poyang Counties, the EWP of Gao’an City also exhibits a decreasing trend with an average annual decrease of 1.529%. The average annual growth rate of EWP in Anyi and Wuning Counties was only 1.210% and 0.141%, respectively; hence, the Xiuhe River basin showed the lowest average annual growth rate of EWP among the seven basins.

The significantly high results of the Super-SBM model, which takes into account the undesired output from a single-stage perspective, indicated that the two-stage Super-NSBM model selected in this study is accurate and reasonable. Moreover, this study can provide a methodological reference for similar studies that measure multistage efficiency.

#### 4.1.2. Spatial Pattern of EWP in the Poyang Lake Area

**Figure 4:**
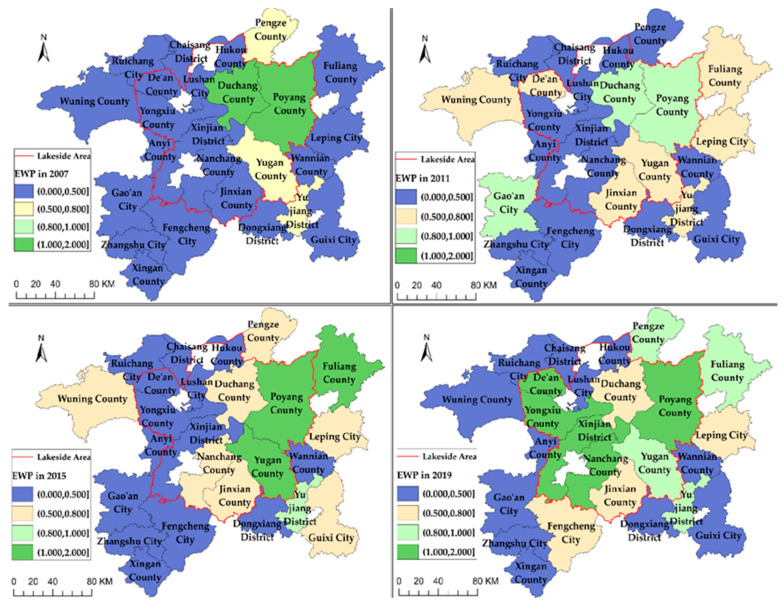
Spatial distribution of EWP in the Poyang Lake area from 2007 to 2019.

Figure 4 reflects the spatial distribution of EWP in the Poyang Lake area at four-time points in 2007, 2011, 2015 and 2019.

The EWP of counties (districts or cities) in the Poyang Lake area was generally at a low level in 2007. Only the EWP of Duchang and Poyang Counties were located on the production front surface. At this time, differences in EWP between the lakeside and peripheral area of the lake are small. The range of high EWP counties (districts or cities) in the Poyang Lake area showed an expanding trend in 2011. EWP of Jinxian County, Fuliang County, Leping City, Wuning County and Yujiang District improved. However, the EWP of Duchang and Poyang Counties dropped below 1. The scope of high EWP counties in the Poyang Lake area continued to expand in 2015. The EWP of counties (districts or cities), such as Nanchang County, Guixi City, Yujiang District and Fuliang County, improved significantly. EWP in the Poyang Lake area improved significantly in 2019 and counties (districts or cities) with low-level EWP are mainly located in the peripheral area of the lake. The EWP of De’an County, Yongxiu County and Xinjian District improved to the efficiency frontier surface and the overall level of EWP of the lakeside area was significantly higher than that of the peripheral area of the lake. Thus, the EWP of the Poyang Lake area shows this spatial distribution pattern—“high EWP counties spread from the lakeside area to the peripheral area of the lake and the EWP of the lakeside area is higher than that of the peripheral area of the lake”—from 2007 to 2019.

### 4.2. Analysis of Sub-Stage Efficiency and Redundancy of EWP in the Poyang Lake Area

This study further analyzed the sub-stage efficiency of EWP and the redundancy of input-output indicators in the Poyang Lake area to open the “black box” of EWP in converting ecological consumption into well-being output.

#### 4.2.1. Sub-Stage Efficiency Analysis of EWP in the Poyang Lake Area

As can be seen from Table 3, the eco-economic efficiency and economic well-being efficiency of the Poyang Lake area from 2007 to 2019 are less than 1, indicating that the coordination of ecological environment, economic development and well-being enhancement in the Poyang Lake area is low. The eco-economic efficiency of the Poyang Lake area was higher than the economic well-being efficiency only in 2007 and its growth rate is low. This result indicated that the low eco-economic efficiency is the primary reason that restricts the improvement of EWP in the Poyang Lake area and the increasing attention to well-being enhancement in the Poyang Lake area yearly alleviates the problem of the well-being level lagging behind economic development. However, the Poyang Lake area must further coordinate the relationship between economy and ecology to promote eco-economy efficiency while improving people’s well-being.

Specifically, the improvement of EWP in the Lakeside area is mainly constrained by the efficiency of economic well-being. Compared with the peripheral area of the lake, the economic level of the lakeside area results in a significantly higher value for eco-economic efficiency because the area is more developed, more technologically advanced and presents stricter environmental protection policies. By contrast, the low EWP in the peripheral area of the lake is mainly due to constraints of eco-economic efficiency. Only the Xinjiang River basin in the region shows higher eco-economic efficiency than economic well-being efficiency. Among the 25 counties (districts or cities), only Poyang County achieved DEA relative effectiveness in both eco-economic and economic well-being efficiencies in 2019. Notably, Dongxiang District’s economic well-being efficiency improves yearly and achieved DEA comparative effectiveness in 2019 despite an eco-economic efficiency of only 0.280. Dongxiang District must improve its eco-economic efficiency to enable coordinated economic, ecological and social development. Jinxian County achieved an effective DEA for economic well-being efficiency but faced the same problem of incompatible economic and environmental development. The eco-economic efficiency of Yogan County, Nanchang County, De’an County, Xinjian District, Yongxiu County and Yujiang District increased beyond the production frontier surface and the efficiency of economic well-being needs further improvement.

#### 4.2.2. Redundancy Analysis of EWP in the Poyang Lake Area

Although some counties achieved an effective DEA for EWP in 2019, the majority still present a low EWP. Therefore, this study selected 2019 as the time point for redundancy analysis. Table 4 demonstrates that the majority of counties in the Poyang Lake area suffer from excessive natural resource input and severe environmental pollution in 2019. This result verifies the previous conclusion that EWP improvement in the Poyang Lake area is mainly constrained by eco-economic efficiency. Meanwhile, over-investment in education spending and science and technology spending is present in most counties. Such overinvestment fails to improve local EWP and is likely to increase local debt. Existing research suggests that many investments aimed at improving ecological well-being come at the cost of increasing the debt of local administrative units [46].

This section focuses on the redundancy of input-output indicators in counties (districts or cities) with low EWP. Lushan City and Hukou County not only spend too much on education but also produce a large amount of carbon and wastewater emissions in the development process and thus have a low EWP. Low land resource use efficiency, high PM2.5 concentration and per capita sewage discharge and a severe shortage of sanitation technicians in Xinkan County are the main reasons for its low EWP. Zhangshu City, Gao’an City and Dongxiang District not only have higher land consumption, water consumption and pollution discharge but also have redundancy in education expenditure. Guixi’s low EWP is caused by low land resource use efficiency and per capita public finance expenditure, while education expenditure and per capita sewage discharge are too high. There is redundancy in education expenditures in Anyi County and the efficiency of water resources utilization, sewage treatment rate and per capita social security and employment expenditures are below average. Wuning County has excessive consumption of land and water resources and the economic output efficiency of education and science and technology expenditure needs to be improved. Chaisang District and Ruichang City are overly redundant in terms of energy consumption, land consumption, water consumption and spending on science and technology. Their PM2.5 concentration and per capita sewage discharge are also much higher than average. In addition, Chaisang District needs to improve its forest cover and sewage treatment rate to contribute to the improvement of environmental well-being.

### 4.3. Spatial Differences Analysis of EWP in the Poyang Lake Area

The analysis results demonstrated that the efficiency of EWP and its sub-stages in the Poyang Lake region differ significantly at the local level. Therefore, this study applied the Dagum Gini coefficient to analyze EWP’s characteristics and sources of spatial differences in the Poyang Lake area.

#### 4.3.1. Overall Spatial Differences of EWP in the Poyang Lake Area

**Figure 5:**
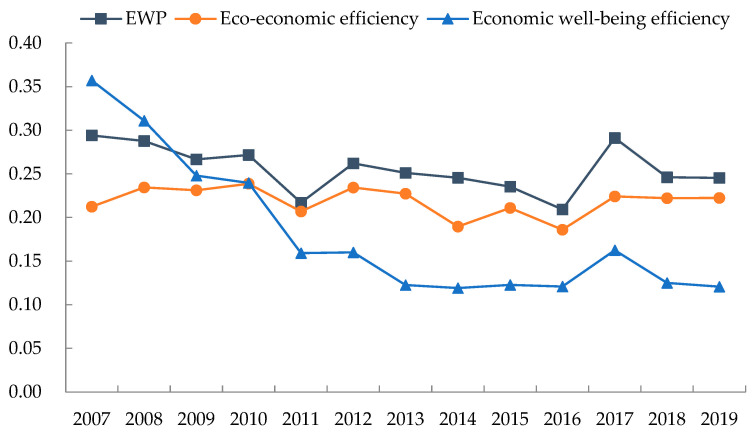
Dagum Gini coefficient of EWP and its phased efficiency in the Poyang Lake area.

As shown in Figure 5, the gradual decrease of spatial differences of EWP indicated that the EWP in the Poyang Lake area generally presents a trend of coordinated development. However, spatial differences of EWP in the Poyang Lake area are still apparent and the Dagum Gini coefficient increased after 2016 and then stabilized at around 0.245. The Dagum Gini coefficient of economic well-being efficiency decreases significantly in terms of efficiency by stages from 0.357 in 2007 to 0.121 in 2019, with an average annual decrease of 8.639%. Combined with the previous analysis, this result indicated that the economic well-being efficiency of the Poyang Lake area shows a trend of convergence and improvement and differences in economic well-being efficiency among counties (districts or cities) gradually decrease. However, the Dagum Gini coefficient of eco-economic efficiency increases from 0.212 in 2007 to 0.222 in 2019, with an average annual increase of 0.386%. This finding demonstrated that spatial differences of EWP in the Poyang Lake area are still significant mainly due to the expansion of spatial differences of eco-economic efficiency. We can then infer that spatial differences of eco-economic efficiency must be reduced to avoid the further expansion of the spatial differences of EWP in the Poyang Lake area.

#### 4.3.2. Spatial Differences Decomposition of EWP in the Poyang Lake Area

We decomposed the spatial differences of EWP in the Poyang Lake area in a basin division according to Equations (3)–(11) to illustrate the characteristics and sources of spatial differences further. Table 5 shows that the Dagum Gini coefficient of intra-basin differences decreases from 0.078 in 2007 to 0.043 in 2019. The contribution rate of intra-basin differences also reduced from 26.67% in 2007 to 17.48% in 2019, thereby suggesting that EWP’s intra-basin differences in the Poyang Lake area are decreasing. However, inter-basin differences are expanding and the Dagum Gini coefficient increases from 0.091 in 2007 to 0.158 in 2019. The contribution rate of inter-basin differences was as high as 64.55% by 2019. This finding exhibited that inter-basin differences are the primary source of EWP spatial differences in the Poyang Lake area.

The annual average values of inter-basin differences in EWP in the Poyang Lake area from 2007 to 2019 are listed in Table 6. Spatial differences in EWP between the Fuhe River basin and Xinjiang River, Poyang Lake, Xiuhe River, Raohe River and Ganjiang River basins reach the maximum, with annual average values of the Dagum Gini coefficient of 0.431, 0.424, 0.369, 0.360 and 0.283, respectively. These spatial differences are caused by the low EWP of Dongxiang District within the Fuhe River basin that only improves from 0.188 in 2007 to 0.280 in 2019. Hence, the gap between the EWP of Fuhe River basin and other basins has been gradually widened. This analysis showed that increasing the EWP of Dongxiang District can effectively reduce the inter-basin differences. We should also note that spatial differences of EWP between the Poyang Lake and Ganjiang River basins and between the Poyang Lake and Xinjiang River basins are also significant. Therefore, introducing cross-regional collaboration policies in the Poyang Lake area in the future is crucial to reduce inter-basin differences and promote the overall coordination and improvement of EWP.

**Figure 6:**
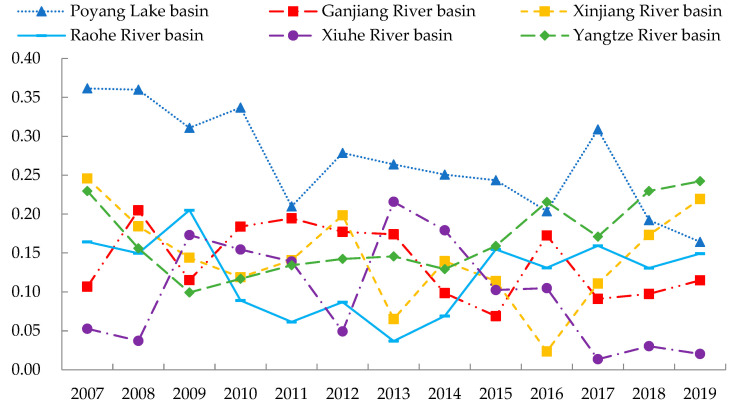
Intra-basin differences of EWP in the Poyang Lake area from 2007 to 2019.

Finally, we analyzed the intra-basin differences of EWP in the Poyang Lake area. The Dagum Gini coefficient of the Fuhe River basin is 0 from 2007 to 2019 given that it only contains Dongxiang District; therefore, the intra-basin differences of EWP in the Fuhe River basin are not shown in Figure 6. Figure 6 presents that the trend of the Dagum Gini coefficient of EWP in the Poyang Lake basin is decreasing with an average annual decrease of 6.360%. Although the average yearly value of the Dagum Gini coefficient in the Xiuhe River basin is only 0.098, it reached the maximum value of 0.216 between 2007 and 2019. The minimum Dagum Gini coefficients of EWP in Xinjiang and Xiuhe River basins from 2007 to 2019 were only 0.065 and 0.037, respectively, but both showed an increasing trend after 2013. Although intra-basin differences of EWP in the Poyang Lake area are narrowing, spatial differences in some basins still show an expanding trend. Therefore, focusing on the elevation of intra-basin spatial differences while reducing inter-basin differences is important.

## 5. Discussion

The sustainable development status of the Poyang Lake area has attracted considerable research attention since the State Council of China approved the Poyang Lake Eco-economic Zone Plan in 2009. However, there is no study in existing research to analyze the sustainable development of the Poyang Lake area through EWP. In this study, a two-stage Super-NSBM model was applied to divide the process from resource input to well-being output into two stages and then analyzed the EWP of the Poyang Lake area. Compared with the study of Long (2019) [30], who also used the model but studied significant cities in China, the EWP of the Poyang Lake area is significantly lower. The reason, on the one hand, is that this study included non-natural resource inputs in the indicator system and the other is that the technological and economic development level of the Poyang Lake area was relatively backward, which leads to low eco-economic efficiency. Like most cities, EWP in the Poyang Lake area is constrained by eco-economic efficiency, suggesting that China still needs to promote the green economic transformation to improve human well-being sustainably [12]. In addition, the measurement results of this study showed that using a two-stage Super-NSBM model is more accurate and reasonable than using the Super-SBM model, which considers undesired outputs from a single-stage perspective. The results of this investigation can provide methodological references for other regional EWP studies with multiple efficiency measures.

Although the EWP of the Poyang Lake area demonstrates an improving trend from 2007 to 2019, the overall level is still low. Most counties have problems of over-input of natural resources and environmental pollution. At the same time, counties should beware of over-investment of funds, which fails to improve EWP significantly and may lead to increased debt. Specifically, each county and region should apply differentiated measures. Lushan City and Hukou County must reduce excessive spending on education, introduce advanced technology and promote the green transformation of industries. Xinkan County should improve land use efficiency, increase the number of health technicians and strictly limit PM2.5 and wastewater emissions from enterprises. Zhangshu City, Gaoan City and Dongxiang District must reduce land and water consumption, minimize education expenditure redundancy and limit pollution discharge. Guixi City should improve land use efficiency and public finance expenditure, reduce education expenditure redundancy and limit wastewater discharge. Chaisang District and Ruichang City must reduce the consumption of various resources and pollution discharge and increase the forest coverage and sewage treatment rate. Anyi County should pay attention to the utilization of water resources and wastewater treatment, improve the efficiency of education expenditures and increase social security and employment expenditures. Wuning County must reduce the consumption of land and water resources and excess spending on education, science and technology.

The differences of EWP and its sub-stage efficiency and indicator redundancy among counties and districts confirmed that EWP in the Poyang Lake area indeed has spatial differences at the local level. The widening spatial differences in eco-economic efficiency are a significant reason why the spatial differences of EWP in the Poyang Lake area are still apparent. Meanwhile, we found that inter-basin differences are the primary source of spatial differences in EWP in the Poyang Lake area. Therefore, the policy focus of the future coordinated development of the EWP region in the Poyang Lake area should be to introduce a cross-regional collaboration mechanism. Regions with advantages in production efficiency, technology level and resource endowment are targeted to help lagging areas to smooth their development mode and realize green economic transformation. The government should also actively help lagging regions increase their income, improve infrastructure construction and social security systems and enhance people’s well-being. Finally, strengthening the construction of ecological civilization within each basin, establishing regional environmental regulatory agencies and establishing long-term cooperation for sustainable development are crucial to avoid intra-basin spatial disparities from widening while narrowing inter-basin differences.

However, this study also has some limitations. Regarding the selection of well-being indicators, due to the lack of survey data on subjective well-being in the Poyang Lake area, we can only select objective well-being indicators. Our future research direction is to investigate residents’ subjective well-being in the Poyang Lake area and then analyze EWP by combining subjective and objective well-being indicators. Besides, applying the system dynamics method to the analysis of EWP in the Poyang Lake area is also worth exploring.

## 6. Conclusions

The main conclusions of this research are as follows.

First, the EWP of the Poyang Lake area shows an improving trend from 2007 to 2019, but the overall level is still low. The EWP of all seven basins failed to achieve DEA effectiveness. The spatial distribution of EWP in the Poyang Lake area shows this apparent pattern—“the high EWP counties spread from the lakeside area to the peripheral area of the lake and the EWP in the lakeside area is higher than that in the peripheral area of the lake.”

Second, there is a complex problem of uncoordinated development between the economy, ecology and society in the Poyang Lake area as a whole. The low eco-economic efficiency mainly constrained the improvement of EWP in the Poyang Lake area and the redundancy of resource input and environmental pollution were more severe in all counties and districts in general. The eco-economic efficiency of the lakeside area is higher and the primary goal is to coordinate the relationship between economy and well-being to promote the efficiency of economic well-being. The redundancy analysis revealed that measures to improve EWP varied across counties.

Third, the spatial differences of EWP in the Poyang Lake area are gradually decreasing and show the overall trend of coordinated development. However, the spatial differences of eco-economic efficiency are progressively expanding, resulting in the spatial differences of EWP in the Poyang Lake area at the local level being still evident. Inter-basin differences are the primary source of spatial differences in the Poyang Lake area’s EWP. Reducing the inter-basin differences of EWP in the Poyang Lake area while avoiding the elevated intra-basin disparities is crucial.

## Figures and Tables

**Table 1:** Evaluation index system of EWP in the Poyang Lake area.

Stage	Category	First Tier	Second Tier	Third Tier Indicators
Stage 1 (Eco-economic efficiency)	Input indicators	Ecological inputs	Energy	Energy consumption per capita
			Land	Built-up area per capita
			Water	Water use per capita
		Nonecological inputs	Human capital	Education expenditure per capita
			Science and technology	Science and technology expenditure per capita
	Undesired output	Environmental pollution	Environmental pollution index	Average PM2.5 concentration, per capita carbon emission and per capita wastewater discharge
Stage 2 (Economic well-being efficiency)	Desired output	Economic output	GDP	GDP per capita
	Output indicators	Comprehensive well-being outputs	Economic well-being index	Urban per capita disposable income and rural disposable income per capita
			Environmental well-being index	Forest coverage rate, sewage treatment rate, per capita energy saving and environmental protection expenditure and per capita carbon dioxide sequestration
			Social well-being index	Number of health technicians per 10,000 people, average years of education, public finance expenditure per capita, social security and employment expenditure per capita and number of employed persons

**Table 2:** Time series trend of EWP by county from 2007 to 2019.

Region	2007	2009	2011	2013	2015	2017	2019	Average Value ^a^	Average Value ^b^	Growth Rate (%)
Yugan County	0.514	0.530	0.713	0.910	1.150	1.931	0.869	0.907	1.544	4.478
Nanchang County	0.164	0.181	0.481	0.489	0.532	0.415	1.020	0.447	0.872	16.453
Lushan City	0.279	0.272	0.324	0.322	0.371	0.331	0.455	0.339	0.591	4.159
De’an County	0.209	0.260	0.610	0.676	0.433	0.669	1.018	0.527	0.833	14.089
Xinjian District	0.227	0.240	0.299	0.237	0.391	0.413	1.002	0.393	0.682	13.183
Yongxiu County	0.234	0.324	0.384	0.350	0.449	0.926	1.023	0.482	0.926	13.067
Hukou County	0.290	0.234	0.306	0.292	0.305	0.283	0.381	0.293	0.492	2.289
Jinxian County	0.449	0.558	0.736	0.678	0.740	0.771	0.734	0.676	1.203	4.189
Duchang County	1.090	0.996	0.815	0.944	0.722	0.776	0.535	0.870	1.316	−5.756
Poyang County	1.044	0.777	0.832	1.004	1.029	0.986	1.034	1.005	1.225	−0.082
Fengcheng City	0.304	0.287	0.365	0.399	0.446	0.475	0.585	0.406	1.013	5.622
Xingan County	0.328	0.323	0.440	0.694	0.436	0.332	0.392	0.444	0.846	1.476
Zhangshu City	0.258	0.380	0.380	0.393	0.443	0.301	0.337	0.378	0.943	2.256
Gao’an City	0.439	0.496	0.881	0.285	0.299	0.335	0.365	0.498	0.949	−1.529
Dongxiang District	0.188	0.228	0.219	0.231	0.247	0.265	0.280	0.239	0.611	3.375
Yujiang District	0.531	0.445	0.528	0.568	0.945	0.593	0.887	0.670	1.113	4.364
Guixi City	0.181	0.246	0.296	0.437	0.594	0.378	0.346	0.390	0.850	5.541
Wannian County	0.185	0.349	0.452	0.663	0.499	0.464	0.459	0.492	0.880	7.865
Leping City	0.376	0.437	0.504	0.624	0.698	0.630	0.562	0.558	1.015	3.417
Fuliang County	0.429	0.853	0.595	0.736	1.011	0.953	0.886	0.793	1.188	6.231
Anyi County	0.306	0.390	0.410	0.417	0.481	0.377	0.354	0.431	0.625	1.210
Wuning County	0.378	0.803	0.726	1.050	0.729	0.398	0.384	0.670	1.178	0.141
Pengze County	0.526	0.321	0.431	0.483	0.545	0.570	0.860	0.535	0.984	4.182
Chaisang District	0.183	0.228	0.220	0.229	0.247	0.246	0.292	0.235	0.378	3.956
Ruichang City	0.287	0.364	0.397	0.451	0.457	0.447	0.411	0.409	0.706	3.046
Poyang Lake basin	0.450	0.437	0.550	0.590	0.612	0.750	0.807	0.594	0.968	4.988
Ganjiang River basin	0.332	0.371	0.517	0.443	0.406	0.361	0.420	0.432	0.938	1.968
Fuhe River basin	0.188	0.228	0.219	0.231	0.247	0.265	0.280	0.239	0.611	3.375
Xinjiang River basin	0.356	0.346	0.412	0.502	0.769	0.485	0.617	0.530	0.981	4.677
Raohe River basin	0.330	0.546	0.517	0.675	0.736	0.682	0.636	0.614	1.028	5.619
Xiuhe River basin	0.342	0.596	0.568	0.733	0.605	0.388	0.369	0.551	0.901	0.635
Yangtze River basin	0.332	0.304	0.349	0.388	0.417	0.421	0.521	0.393	0.689	3.827

Note: Average value ^a^ is the measurement result of this study using the two-stage Super-NSBM model; Average value ^b^ is the measurement result of using the Super-SBM model considering undesired output.

**Table 3:** Subphase efficiency of EWP in the Poyang Lake area from 2007 to 2019.

Region	2007		2011		2015		2019		2007–2019	
	Stage1	Stage2	Stage1	Stage2	Stage1	Stage2	Stage1	Stage2	Stage1	Stage2
Yugan County	1.000	0.346	0.919	0.620	1.118	1.063	1.001	0.757	1.024	0.757
Nanchang County	0.359	0.126	0.633	0.396	0.626	0.564	1.108	0.837	0.580	0.456
Lushan City	0.654	0.316	0.384	0.608	0.404	0.798	0.514	0.747	0.437	0.603
De’an County	0.340	0.336	0.614	0.951	0.448	0.901	1.026	0.984	0.574	0.754
Xinjian District	0.410	0.185	0.382	0.470	0.556	0.468	1.048	0.914	0.537	0.431
Yongxiu County	0.469	0.229	0.444	0.674	0.484	0.830	1.089	0.877	0.570	0.625
Hukou County	0.552	0.258	0.382	0.498	0.376	0.572	0.470	0.545	0.391	0.476
Jinxian County	1.026	0.212	0.985	0.526	0.896	0.666	0.734	1.000	0.924	0.552
Duchang County	1.032	1.112	0.816	0.986	0.720	1.007	0.671	0.580	0.886	0.926
Poyang County	0.941	1.221	0.825	1.016	1.030	0.999	1.008	1.053	0.961	1.072
Fengcheng City	0.427	0.401	0.401	0.774	0.506	0.775	0.625	0.860	0.471	0.691
Xingan County	0.497	0.404	0.529	0.629	0.520	0.660	0.496	0.536	0.573	0.550
Zhangshu City	0.462	0.213	0.472	0.550	0.556	0.556	0.415	0.566	0.539	0.463
Gao’an City	0.752	0.301	1.066	0.688	0.311	0.922	0.428	0.646	0.594	0.701
Dongxiang District	0.358	0.256	0.249	0.707	0.263	0.870	0.280	1.000	0.277	0.752
Yujiang District	1.000	0.362	0.636	0.616	0.939	1.011	1.013	0.775	0.808	0.698
Guixi City	0.374	0.118	0.411	0.319	0.727	0.551	0.466	0.420	0.516	0.410
Wannian County	0.395	0.307	0.528	0.695	0.559	0.767	0.523	0.719	0.570	0.690
Leping City	0.813	0.234	0.651	0.515	0.789	0.772	0.594	0.881	0.732	0.590
Fuliang County	0.429	1.000	0.595	1.000	1.042	0.941	0.934	0.900	0.844	0.898
Anyi County	0.614	0.227	0.511	0.545	0.572	0.642	0.426	0.614	0.562	0.526
Wuning County	0.615	0.324	0.811	0.772	0.760	0.925	0.421	0.802	0.736	0.791
Pengze County	0.523	1.014	0.439	0.959	0.562	0.934	0.913	0.882	0.575	0.888
Chaisang District	0.431	0.213	0.279	0.507	0.294	0.638	0.336	0.691	0.316	0.518
Ruichang City	0.421	0.487	0.437	0.779	0.505	0.765	0.458	0.769	0.488	0.743
Poyang Lake area	0.596	0.408	0.576	0.672	0.622	0.784	0.680	0.774	0.619	0.662
Lakeside area	0.678	0.434	0.638	0.675	0.666	0.787	0.867	0.829	0.688	0.665
Peripheral area of the lake	0.541	0.391	0.534	0.670	0.594	0.782	0.555	0.737	0.573	0.661
Poyang Lake basin	0.678	0.434	0.638	0.675	0.666	0.787	0.867	0.829	0.688	0.665
Ganjiang River basin	0.534	0.330	0.617	0.660	0.473	0.728	0.491	0.652	0.544	0.601
Fuhe River basin	0.358	0.256	0.249	0.707	0.263	0.870	0.280	1.000	0.277	0.752
Xinjiang River basin	0.687	0.240	0.524	0.467	0.833	0.781	0.739	0.597	0.662	0.554
Raohe River basin	0.546	0.513	0.591	0.737	0.797	0.827	0.683	0.833	0.715	0.726
Xiuhe River basin	0.615	0.275	0.661	0.659	0.666	0.783	0.424	0.708	0.649	0.659
Yangtze River basin	0.458	0.572	0.385	0.748	0.454	0.779	0.569	0.781	0.460	0.716

**Table 4:** Redundancy of input-output indicators of each county (district or city) in the Poyang Lake area in 2019.

Region	R_1_	R_2_	R_3_	N_1_	N_2_	I_1_	U_1_	D_1_	D_2_	D_3_
Yugan County	−0.038	0.000	−0.152	−70.454	−5.729	−1900	0.000	0.000	0.058	0.149
Nanchang County	0.000	0.000	0.000	0.000	0.000	−18,930	0.000	−0.115	0.000	0.000
Lushan City	−0.446	−9.676	−2.670	−546.536	−189.813	−6030	−0.168	0.000	0.216	0.000
De’an County	0.000	0.000	0.000	0.000	0.000	−4010	0.000	0.000	0.000	−0.087
Xinjian District	0.000	0.000	0.000	0.000	0.000	−4420	0.000	−0.014	0.000	0.000
Yongxiu County	0.000	0.000	0.000	0.000	0.000	−9890	0.000	0.000	−0.095	−0.003
Hukou County	−1.759	−7.082	−11.418	−1082.392	−136.320	−33,560	−0.230	0.000	0.100	0.000
Jinxian County	−0.001	−4.500	−4.855	−141.207	−10.499	0.000	−0.050	0.000	0.000	0.000
Duchang County	−0.221	19.259	−2.452	−66.783	−135.124	−10,050	−0.017	0.018	0.000	0.050
Poyang County	0.000	0.000	0.000	22.910	0.000	−110	0.000	−0.035	−0.024	−0.025
Fengcheng City	−0.307	−6.861	0.000	−215.285	−127.771	−2090	−0.087	0.000	0.000	0.110
Xingan County	−0.260	−15.426	−9.462	−412.562	−179.506	−15,870	−0.168	0.000	0.106	0.190
Zhangshu City	−1.121	−18.671	−13.809	−1287.514	−68.342	−25,280	−0.189	0.000	0.148	0.000
Gao’an City	−1.687	−11.080	−10.217	−585.006	−233.354	−17,250	−0.150	0.000	0.000	0.041
Dongxiang District	−1.866	−33.874	−15.675	−995.799	−356.135	0.000	−0.202	0.000	0.000	0.000
Yujiang District	−0.006	−0.094	−0.060	0.000	−5.875	−1670	−0.002	0.063	0.236	0.020
Guixi City	−1.468	−22.379	−5.032	−1042.078	−150.479	−40,510	−0.125	0.000	0.000	0.135
Wannian County	−0.421	−13.103	−6.325	−334.881	−172.055	−10,170	−0.073	0.000	0.010	0.013
Leping City	−0.383	0.000	−4.315	−705.835	−104.048	−3990	−0.062	0.000	0.013	0.000
Fuliang County	−0.507	0.000	−0.366	−31.875	−5.048	−1630	−0.015	0.000	0.000	0.103
Anyi County	−0.611	−3.398	−30.925	−636.309	−42.688	−10340	−0.246	0.000	0.179	0.118
Wuning County	−0.952	−13.437	−11.980	−665.343	−222.333	−7400	−0.087	0.000	0.000	0.033
Pengze County	−0.211	0.000	−2.154	−136.819	−32.610	−3800	−0.023	0.087	0.000	0.000
Chaisang District	−4.104	−20.921	−14.422	400.196	−199.126	−7660	−0.539	0.000	0.261	0.000
Ruichang City	−2.298	−14.586	−9.209	−254.500	−430.332	−9440	−0.132	0.000	0.125	0.000

Note: R_1_, R_2_ and R_3_ represent per capita energy consumption, per capita built-up area and per capita water supply, respectively; N_1_ and N_2_ represent per capita education expenditure and per capita science and technology expenditure, respectively; I1 represents per capita GDP; U_1_ represents environmental pollution index; D_1_, D_2_ and D_3_ represent economic, environmental and social well-being indices, respectively.

**Table 5:** Spatial differences decomposition of EWP in the Poyang Lake area from 2007 to 2019.

Year	*G*	*G_w_*	Contribution Rate (%)	*G_nb_*	Contribution Rate (%)	*G_t_*	Contribution Rate (%)
2007	0.294	0.078	26.67	0.091	30.95	0.125	42.38
2008	0.288	0.075	25.97	0.074	25.66	0.139	48.36
2009	0.267	0.061	23.05	0.119	44.67	0.086	32.28
2010	0.272	0.066	24.47	0.117	35.78	0.108	39.75
2011	0.217	0.047	21.50	0.085	39.35	0.085	39.15
2012	0.262	0.055	20.95	0.129	49.10	0.078	29.95
2013	0.251	0.054	21.50	0.120	47.93	0.077	30.57
2014	0.246	0.052	21.01	0.130	52.77	0.064	26.22
2015	0.235	0.050	21.03	0.128	54.45	0.058	24.52
2016	0.209	0.043	20.75	0.097	46.40	0.069	32.86
2017	0.291	0.072	24.61	0.168	57.52	0.052	17.87
2018	0.246	0.045	18.36	0.147	59.70	0.054	21.95
2019	0.245	0.043	17.48	0.158	64.55	0.044	17.97

**Table 6:** Annual average values of inter-basin differences of EWP in the Poyang Lake area from 2007 to 2019.

Region	*G_jh_*	Region	*G_jh_*	Region	*G_jh_*
1–2	0.282	2–4	0.226	3–7	0.247
1–3	0.424	2–5	0.234	4–5	0.184
1–4	0.257	2–6	0.190	4–6	0.235
1–5	0.237	2–7	0.196	4–7	0.216
1–6	0.276	3–4	0.360	5–6	0.201
1–7	0.296	3–5	0.431	5–7	0.253
2–3	0.283	3–6	0.369	6–7	0.252

Note: 1, 2, 3, 4, 5, 6 and 7 represent Poyang Lake basin, Ganjiang River basin, Fuhe River basin, Xinjiang River basin, Raohe River basin, Xiuhe River basin and Yangtze River basin, respectively.

## Data Availability

The data presented in this study are available from the corresponding author.
